# Black‐Tailed Godwits Eat Animal and Plant Material at a Key Non‐Breeding Site at the Overlap of Two Asian Flyways

**DOI:** 10.1002/ece3.74479

**Published:** 2026-10-18

**Authors:** Delip K. Das, Naim Khandakar, Md. Soab Ali, Wouter M. G. Vansteelant, El‐Hacen M. El‐Hacen, Theunis Piersma

**Affiliations:** ^1^ Delta Sentinels Lab, Department of Zoology, Faculty of Life and Earth Sciences Jagannath University Dhaka Bangladesh; ^2^ BirdEyes, Centre for Global Ecological Change at the Faculties of Science & Engineering and Campus Fryslân University of Groningen Leeuwarden the Netherlands; ^3^ Conservation Ecology Group, Groningen Institute for Evolutionary Life Sciences University of Groningen Groningen the Netherlands; ^4^ Theoretical and Computational Ecology, Institute for Biodiversity and Ecosystem Dynamics University of Amsterdam Amsterdam the Netherlands; ^5^ Department of Coastal Systems NIOZ Royal Netherlands Institute for Sea Research Texel the Netherlands

**Keywords:** bay of Bengal, central Asian flyway, droppings analysis, east Asian–Australasian flyway, non‐breeding diet, shorebirds

## Abstract

Dietary knowledge of migratory birds along their migration routes is fundamental for understanding ecological and evolutionary aspects of bird migration, how migrants select and repeatedly use specific non‐breeding areas, accumulate fuel for migration and breeding, and assess carrying capacity at key sites in the migratory network. Local changes in food availability and foraging habitat have been shown to contribute to population declines in migratory shorebirds. Nevertheless, the diet of many migratory bird populations remains unknown at key migratory sites. We address this knowledge gap for Black‐tailed Godwits ( *Limosa limosa* ) by exploring their diet in the Ganges–Brahmaputra–Meghna delta in the Bay of Bengal, a key non‐breeding site overlapping the Central Asian and East Asian–Australasian flyways. We assessed how benthic prey availability changed during their non‐breeding period based on 600 mud samples collected from 120 foraging locations between October 2018 and March 2019. In mid‐January 2019, we determined godwit diets by analyzing undigested remains of 15 feces and 15 pellets collected from roosting areas, and compared dietary composition with benthic prey availability from samples collected concurrently. Polychaetes and oligochaetes were the most abundant benthic groups in the mud, but we did not find them in the droppings. We observed intra‐seasonal changes in abundance and body size of bivalvia, decapoda, and gastropoda, which were the main animal foods found in droppings and mud samples, and were positively selected by godwits based on availability compared to other benthic prey. We also found plant materials, rice kernels, and rhizomes of *Porteresia coarctata*, in one‐third of the droppings. This finding extends previous evidence of godwits' herbivory in the East‐Atlantic flyway to Asian flyways. Scaling up our efforts to systematic monitoring could help address conservation questions, such as the impact of shellfish harvesting on the carrying capacity of the world's largest river delta for shorebirds.

## Graphical abstract

Food resources are essential for understanding habitat use, migration strategies, and population dynamics of migratory shorebirds, yet dietary information for Black‐tailed Godwits in Asian flyways remains poorly known. We provide the first assessment of their non‐breeding diet in the Ganges–Brahmaputra–Meghna delta, showing seasonal changes in prey availability, selective consumption of bivalves, gastropods, and decapods, and evidence of herbivory extending previous findings to Asian flyways.

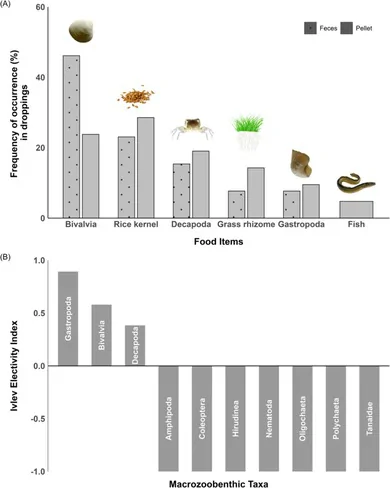

## 1 Introduction

Migratory birds track seasonally available food and other resources across the globe (Winkler et al. 2016). They use different kinds of habitats in various locations throughout their annual cycle, switching between a variety of food resources available in each habitat and required at different stages of the year. Non‐breeding sites are particularly important because migratory birds can spend more than half a year, relying on local environmental conditions and food resources to nourish, survive, and prepare for the next migration (Marra et al. 2015; Piersma et al. 2022). Understanding what food resources are used along the migration flyways is an essential first step to formulate evolutionary explanations of bird migration (van de Kam et al. 2004; Piersma et al. 2022). Diet information is fundamental in understanding how migrants select and repeatedly use specific non‐breeding areas where predictable prey support survival during the non‐breeding season (Penning et al. 2026) and accumulate fuel for migration and breeding (Marra et al. 2015; Chan et al. 2023). In addition, quantifying diet composition is useful to assess (changes in) the carrying capacity at key sites in the migratory network, particularly in coastal ecosystems where migratory shorebirds rely heavily on macrozoobenthic prey communities (Piersma et al. 1993; Correia et al. 2023). The abundance, diversity, and accessibility of benthic organisms determine how many individuals a site can support and whether habitat degradation or environmental change may lead to food limitation (Piersma et al. 1993; Peng 2024). Local changes in food availability and foraging habitat have been shown to contribute to population declines in migratory shorebirds (Studds et al. 2017).

Despite the importance, for the majority of shorebirds, knowledge on diets along the migration route is lacking, including species of conservation concern. For example, Black‐tailed Godwits ( *Limosa limosa* ; Figure 1), hereafter referred to as godwits, are long‐distance migratory birds, listed as Near Threatened because of their declining population (BirdLife International 2026). Four subspecies (*L. l. limosa, L. l. islandica, L. l. bohaii*, and, *L. l. melanuroides*) of godwits have been recognized. Populations of these subspecies are widely distributed across Eurasia, migrate based on their breeding and non‐breeding areas along the five shorebird flyways–East Atlantic Flyway, Black Sea–Mediterranean Flyway, West Asian–East African Flyway, Central Asian Flyway, and East Asian–Australasian Flyway (BirdLife International 2026). Diet information is available only in few key stopovers and non‐breeding locations of the East Atlantic Flyway (Moreira 1994; Tréca 1994; Gill et al. 2001; Green and Sánchez 2006; Sánchez et al. 2006; Lourenço and Piersma 2008; Hayhow 2009; Estrella and Masero 2010; Catry et al. 2012; Robin et al. 2013, 2015), but lacks in the rest of the flyways including two partially overlapping Asian flyways: Central Asian and East Asian–Australasian flyways.

**FIGURE 1:**
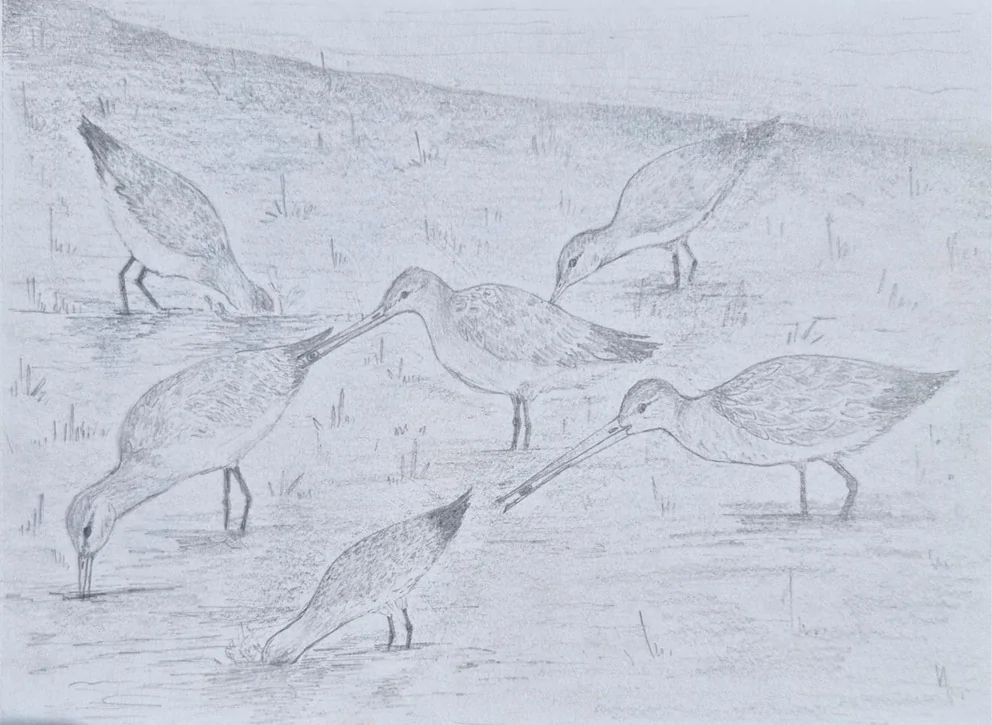
Illustration of Black‐tailed Godwits foraging on a mudflat. Drawing by Ritika Chatterjee.

In Asian flyways, recent studies confirmed the occurrence of populations of three subspecies of godwits (*limosa, melanuroides* and *bohaii*–Zhu et al. 2021; Zhu et al. 2023; Das et al. 2025). The finding that during the non‐breeding period all three co‐occur in the world's largest river delta in Bangladesh (Das et al. 2025) is exciting from an eco‐evolutionary perspective. For example, because body size differs between the three subspecies and their sexes, larger and longer‐billed individuals may access prey buried deeper in the mud as well as forage in deeper waters, while smaller and short‐billed individuals can specialize in prey in the upper layer of mudflats and in shallow waters or different food resources (Das et al. 2025; Zhu et al. 2020). This can lead to different prey selection, foraging strategies, and spatial distribution (Alves et al. 2013; Zhu et al. 2026). Understanding whether this is the case across the sites is fundamental for insights into ecological adaptations and selection pressure of subspecies and sex across flyways. The first step starts with knowing the diet characteristics of these godwits, which is still lacking.

To address this knowledge gap, we made a first exploration of the diets of godwits in Nijhum Dweep National Park, one of the most important non‐breeding sites for godwits in Bangladesh (Das et al. 2022). We surveyed changes in the availability of macrozoobenthic taxa in intertidal soft‐sediment foraging areas throughout the non‐breeding period of 2018–2019 and assessed use and selectivity of godwits for different prey types by sampling regurgitated pellets and feces in the middle of the non‐breeding season.

## 2 Methods

### 2.1 Study Area

The Nijhum Dweep National Park (22,003′51.4″ N, 91000′03.4″ E)—an Important Bird and Biodiversity Area (IBA) recorded 161 bird species (Khandakar et al. 2026)—is located in the Meghna Estuary of the Bay of Bengal at the south coast of Bangladesh (Figure 2). Spanning an area of 16,352 ha, the park includes a variety of habitats, such as mangrove forests, intertidal mudflats, sandy beaches, brackish marshes, and agricultural land. Approximately 8000–9000 godwits forage in the intertidal mudflats of the park (Das et al. 2022). However, the park's land cover is constantly changing, particularly due to the dynamics of the intertidal systems. Geomorphological processes in coastal ecosystems such as erosion, sediment deposition, shifting sandbars, and evolving channels shape the landscape, leading to continuous land transformation (Brammer 2014; Emran et al. 2015; Hossain et al. 2016). In addition, human activities such as land reclamation by planting mangroves (Das et al. 2022) and conversion of land for rice cultivation (Islam 2021) contribute to land cover change. The region has a tropical monsoon climate with high humidity and abundant rainfall, ranging from 1500 to 2000 mm annually, mostly during the monsoon from June to September (Rahman et al. 2019; Kabir et al. 2020).

**FIGURE 2:**
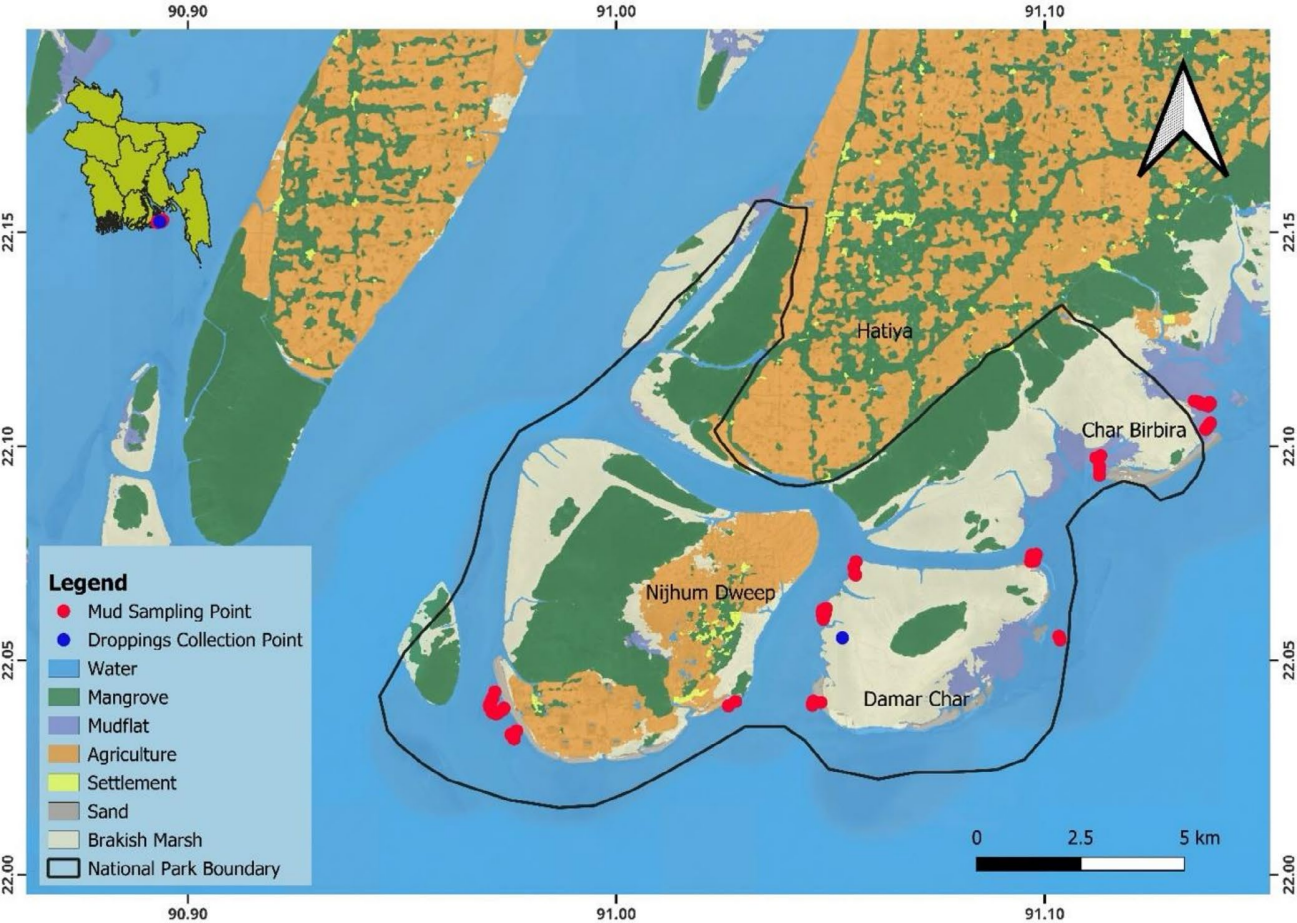
Map of the study area indicating mud sampling points (red circles) at foraging sites and droppings collection point (blue circle) at roosting area of the Black‐tailed Godwit in Nijhum Dweep National Park, Bangladesh.

### 2.2 Macrozoobenthic Sampling and Processing

To investigate diversity, abundance, and size of prey available to godwits in intertidal mudflats of the Ganges–Brahmaputra–Meghna delta, we sampled mud from foraging locations of godwits. First, we identified a location where we observed ≥ 150 godwits foraging during low tide. Second, we walked to the location and collected five mud samples randomly within the area where godwits were observed foraging, using a metal quadrat corer (5 × 5 cm, 15 cm depth). Sampling points were selected without targeting particular sediment types or other visible habitat features. Third, we immediately rinsed mud samples with seawater through a 600‐μm sieve. Finally, we preserved filtered organisms in a labeled vial filled with 70% ethanol. From October 2018 to March 2019, we sampled 120 foraging locations distributed across the park and collected 600 mud samples (Table S1). We grouped eight mud samples collected on April 2, 2019, with the March samples for analysis because they were originally scheduled for March but were delayed by 2 days due to weather and field logistics.

In the laboratory, we counted numbers of individual prey items, took photos of representative individuals using a mounted mobile phone camera to a compound microscope (6× and 10×), and measured the body length of every specimen. Then, we identified benthic items to the lowest possible taxonomic level using the microscope and taxonomic books from the region (Siddiqui et al. 2007; Kabir et al. 2008, 2009; Hossain et al. 2014).

### 2.3 Diet Sampling and Processing

On 15 January 2019 in Damar Char (Figure 2), in the middle of the non‐breeding season, a roosting flock of godwits was observed with a telescope until their departure. When departed, the observer kept an eye on the area and directed another team member to the place to search for feces and pellets to minimize the risk of collecting materials from other species or previously deposited material. With a spoon and tweezers, we collected 15 feces and 15 regurgitated pellets from the substrate and preserved them separately in 70% alcohol. The samples were stored in a freezer before further analysis.

In the laboratory, we thawed the samples and disaggregated them in a petri dish, then sieved them using a tea‐strainer under a continuous stream of water. We categorized the undigested prey remains by taxon. We identified undigested remains to the lowest possible taxonomic level. It is well established that the traditional method of analyzing undigested remains under the microscope could miss digested soft‐bodied prey species (Perez‐Hurtado et al. 1997; Penning et al. 2022). Still, it is a useful approach, especially in contexts where only low‐cost methods are feasible, as was the case in our study.

### 2.4 Data Analysis

The macrozoobenthic composition found in intertidal mudflats was described by frequency of occurrence (presence/absence of taxa in the samples) and assessed by their relative abundance and density. The relative abundance of a taxon was calculated by dividing total individuals of that taxon by the total number of individuals of all taxa. Benthic density of a taxon (individuals per square meter) across all samples was calculated by dividing total individuals of that taxon by the sampling area. Nine of the 600 sediment cores yielded no macrozoobenthic taxa. These cores likely represent sampling failures due to sediment loss during sieving of the surficial layer, rather than genuine faunal absence, and were therefore excluded from further analyses.

To understand the overall seasonal stability of benthic community composition during the non‐breeding season, monthly composition was assessed using non‐metric multidimensional scaling (nMDS) based on Bray–Curtis dissimilarities, and differences among months were tested using PERMANOVA. The relative abundance of macrozoobenthic taxa was first Hellinger transformed using the function *decostand* from the vegan package in R (Oksanen et al. 2018) followed by the calculation of dissimilarity distances using the Bray‐Curtis equation. The resulting dissimilarity matrix was used for analysis of variance of species assemblage between months, using the *adonis2* function for PERMANOVA and nMDS. The dissimilarity matrix was also used to perform a similarity percentage (SIMPER) analysis to distinguish between the effects of each macrozoobenthic taxon in explaining their contribution to the variation in a specific month during the non‐breeding season and thus determine their importance.

The diet composition found in godwits' droppings was described by frequency of occurrence (presence/absence of undigested remains). The frequency of occurrence is simply the frequency at which a food item occurred in the samples expressed as a percentage of all samples. To determine godwits' selection of macrozoobenthic taxa from droppings and mud samples of January 2019, we used Ivlev Electivity Index (*E*) for each prey taxon (Ivlev 1961):

$$E=\frac{\left(P_{d}-P_{a}\right)}{\left(P_{d}+P_{a}\right)}$$

where *P*_d_ is the proportion of a macrozoobenthic taxon in droppings, and *P*_a_ is the proportion of that taxon in benthic sample. The index ranges from −1 (complete avoidance) to +1 (strong selection), with value 0 indicating selection proportional to availability. Across the non‐breeding season, to understand the change in abundance and body size of the macrozoobenthic taxa found in godwits droppings, The relative abundance and body size over the months were fitted for a linear regression using *LOESS* function in package ggplot2 (Wickham 2016). All analyses were conducted in R (version 4.4.1).

## 3 Results

In the mud, we found 10 macrozoobenthic taxa (Figure 3). Of these, polychaetes, oligochaetes, bivalves, gastropods, and decapods were considered potential prey for godwits based on the literature and therefore highlighted the results of these five taxa. The frequency of occurrence (Figure 3A) was high for polychaetes and oligochaetes (79% and 73% respectively), moderate for bivalves and decapods (49% and 39% respectively), and low for gastropods (9%). Likewise, the relative abundance (Figure 3B) and density (Figure 3C) were high for polychaetes and oligochaetes, moderate for bivalves and decapods, and low for gastropods.

**FIGURE 3:**
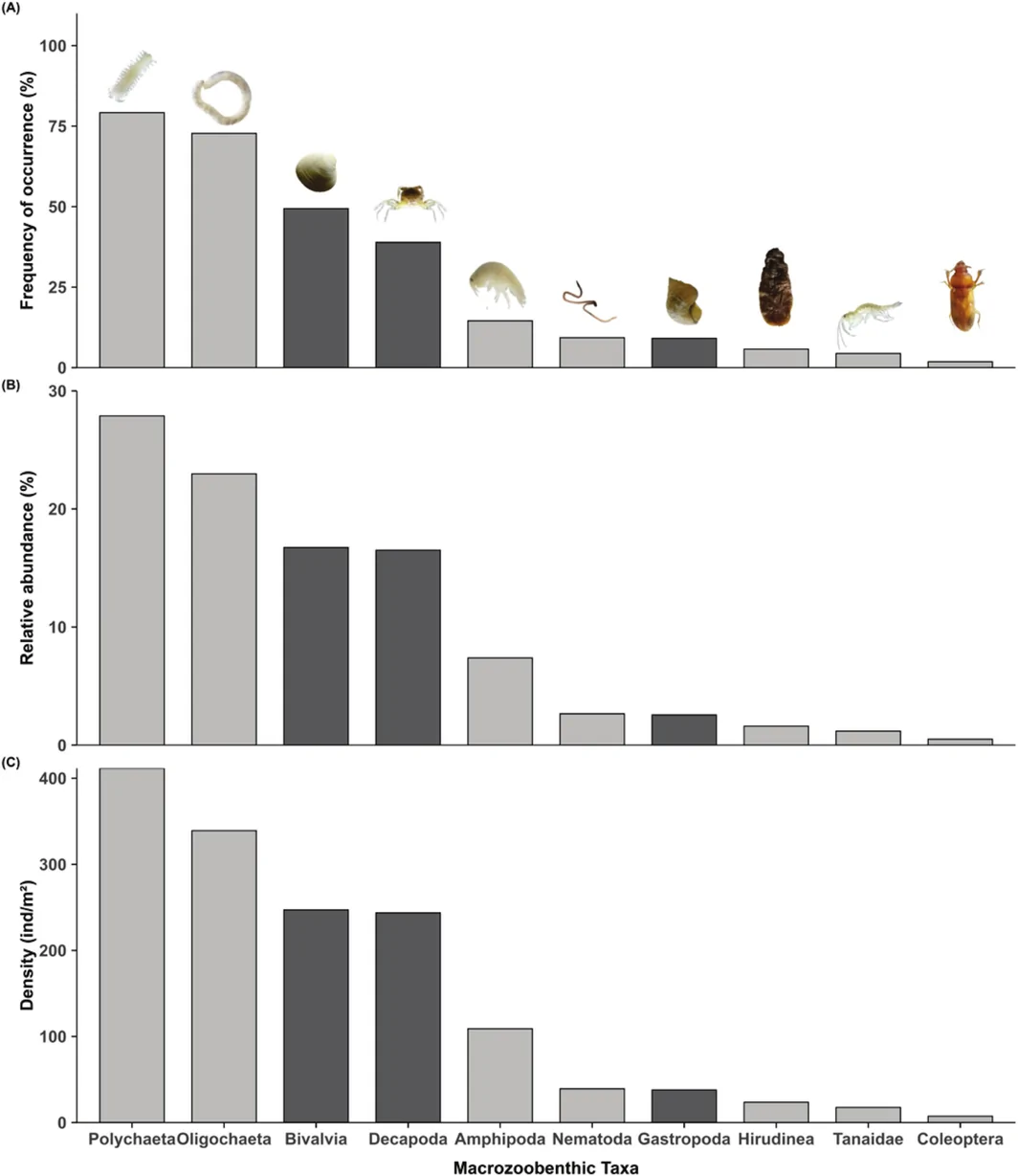
Composition of macrozoobenthic taxa based on mud samples, showing frequency of occurrence (A), relative abundance (B), and density (ind/m^2^) (C) to understand prey diversity and abundance in intertidal mudflats available to godwits. Polychaeta and Oligochaeta showed the highest frequency of occurrence, relative abundance, and densities, followed by Bivalvia and Decapoda. The macrozoobenthic taxa in dark gray represent the groups found in the droppings of Black‐tailed Godwits.

The relative abundance and body size of these benthic groups showed distinct intra‐seasonal patterns (Figure 4). The abundance of bivalves and decapods gradually increased over the sampling period, rising from relatively low values in October–November to the highest levels in February, after which decapods slightly declined in March. In contrast, gastropods exhibited a declining pattern throughout the season (Figure 4a). The body size of bivalves steadily decreased from October to March, but increased for decapods from November to January and then remained stable (Figure 4b). Gastropods showed relatively stable body size during the study period. Body size of polychaetes and oligochaetes also varied seasonally and were much larger‐bodied than hard‐shelled bivalves, decapods, and gastropods (Figure 4b).

**FIGURE 4:**
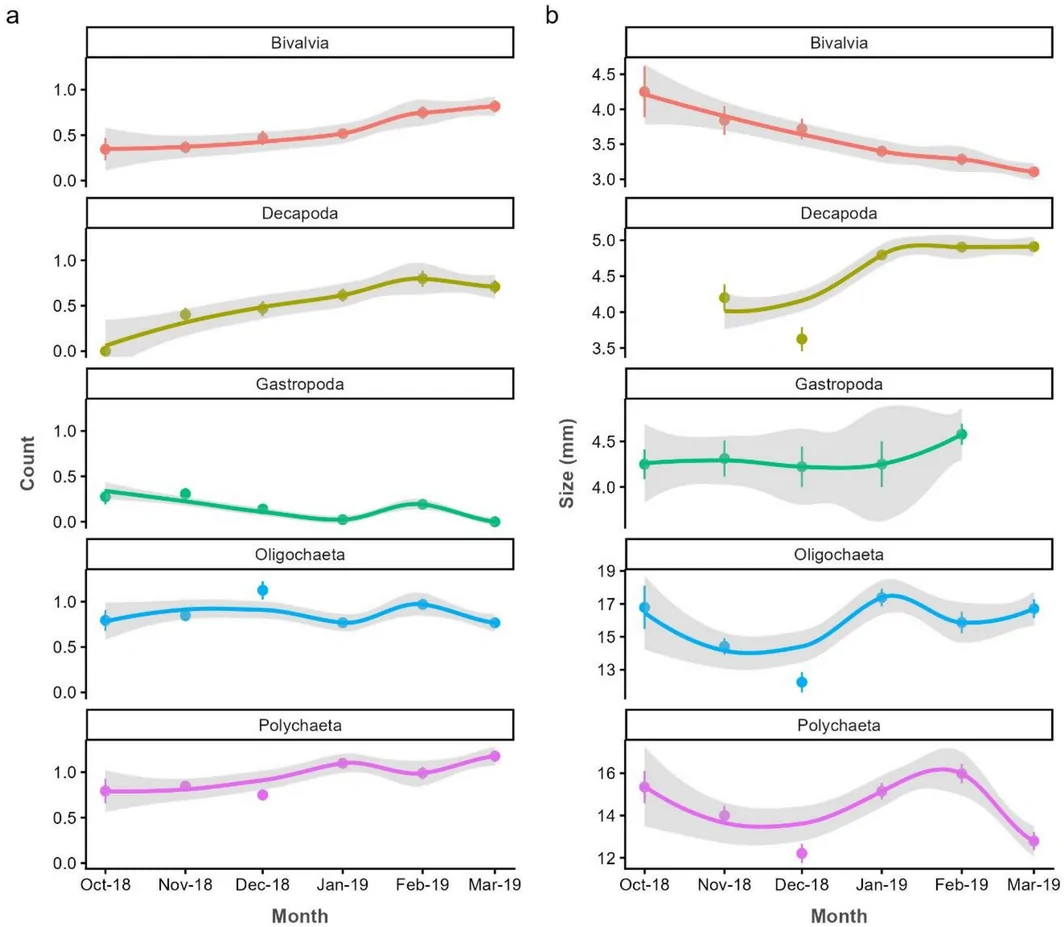
Seasonal variation of five macrozoobenthic taxa sampled from October 2018 to March 2019: (a) relative abundance and (b) body size. Points indicate mean count and size per sampling month and error bars represent ± SE. Solid lines show LOESS‐smoothed trends with 95% confidence intervals (gray).

Despite intra‐seasonal changes in the abundance and body size of benthic taxa, the overall macrozoobenthic community composition showed only moderate temporal variation during the non‐breeding period. The nMDS ordination (3‐dimensional solution, stress = 0.11) showed substantial overlap among monthly samples, indicating broadly similar community composition across months (Figure 5). PERMANOVA nevertheless detected a significant effect of month on community composition (*F* = 7.40, *R* ^2^ = 0.059, *p* = 0.001), although month explained only 5.9% of the total variation in benthic assemblage structure. Homogeneity of multivariate dispersion did not differ significantly among months (PERMDISP, *p* = 0.295), suggesting that the observed differences reflected compositional change rather than unequal within‐month variability. Pairwise PERMANOVA indicated that community differences were generally stronger between early (October–December) and later (January–March) months of the season than among adjacent months within early and late period (Table S2). SIMPER analysis further indicated that Polychaeta, Oligochaeta, Bivalvia, and Decapoda contributed most consistently to temporal dissimilarities in benthic community composition, whereas Gastropoda contributed comparatively less.

**FIGURE 5:**
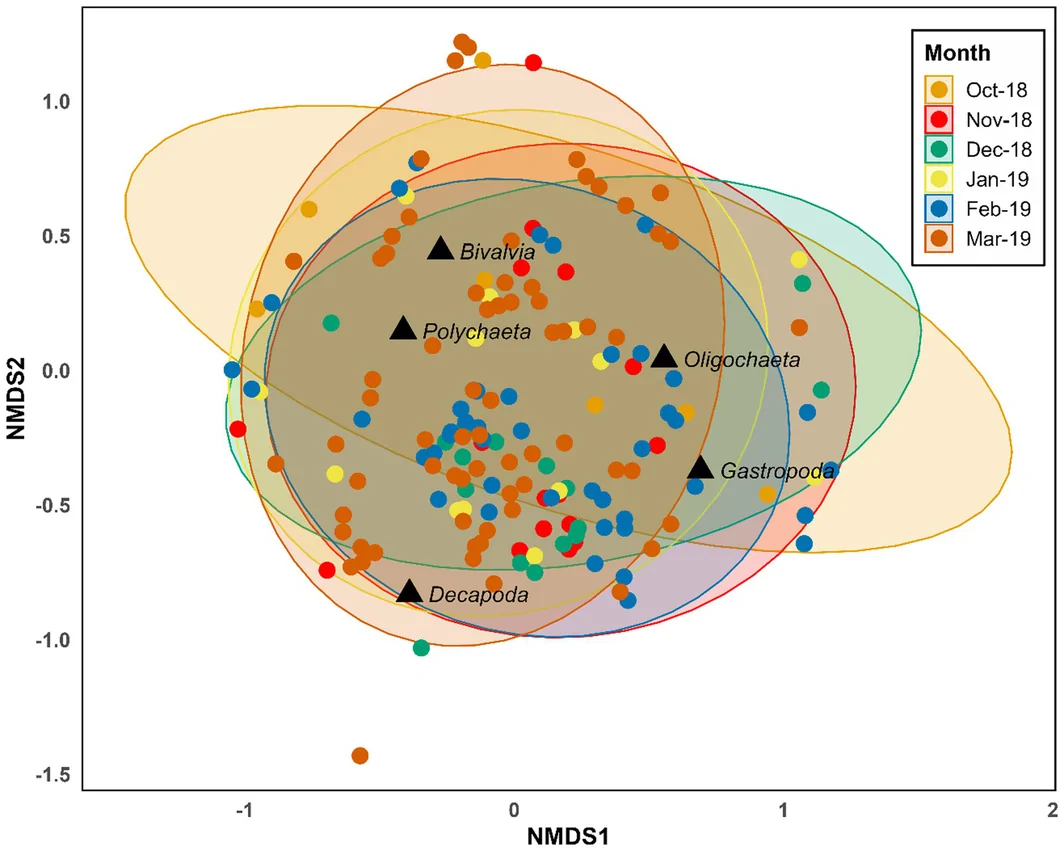
Non‐metric multidimensional scaling (NMDS) ordination showing temporal variation in benthic community composition across sampling months based on Bray–Curtis dissimilarities of Hellinger‐transformed abundance data (3‐dimensional solution, stress = 0.11). Each point represents a sample, with distances between points reflecting relative differences in benthic assemblage composition. Monthly samples showed substantial overlap, indicating broadly similar community structure throughout the non‐breeding season despite significant but weak temporal differences detected by PERMANOVA. Taxa contributing most consistently to temporal dissimilarities included Polychaeta, Oligochaeta, Bivalvia, Decapoda, and Gastropoda.

In godwits' droppings, we found undigested remains of both plant and animal food items (Figure 6A). The plant items comprised rice kernels and rhizomes of Uri Grass *Porteresia coarctata*. Rice kernels occurred in 23% and 29% of feces and pellets respectively. The rhizomes occurred more in pellets (14%) than in feces (8%). The animal items comprised bivalves, decapods, gastropods, and fish. Frequency of occurrence of bivalves was highest, 46% in fecal samples and 24% in pellets. Gastropods occurred relatively less, in 8% of feces and 10% of pellets. Decapods occurred in 15% of feces and 19% of pellets. Fish remains were found only in pellets (5%). Among macrozoobenthic taxa found in the mud in January 2019, we found strong positive selection of gastropods (Ivlev electivity index, *E* = 0.90, Figure 6B) and bivalves (*E* = 0.58), and moderate selection of decapods (*E* = 0.39).

**FIGURE 6:**
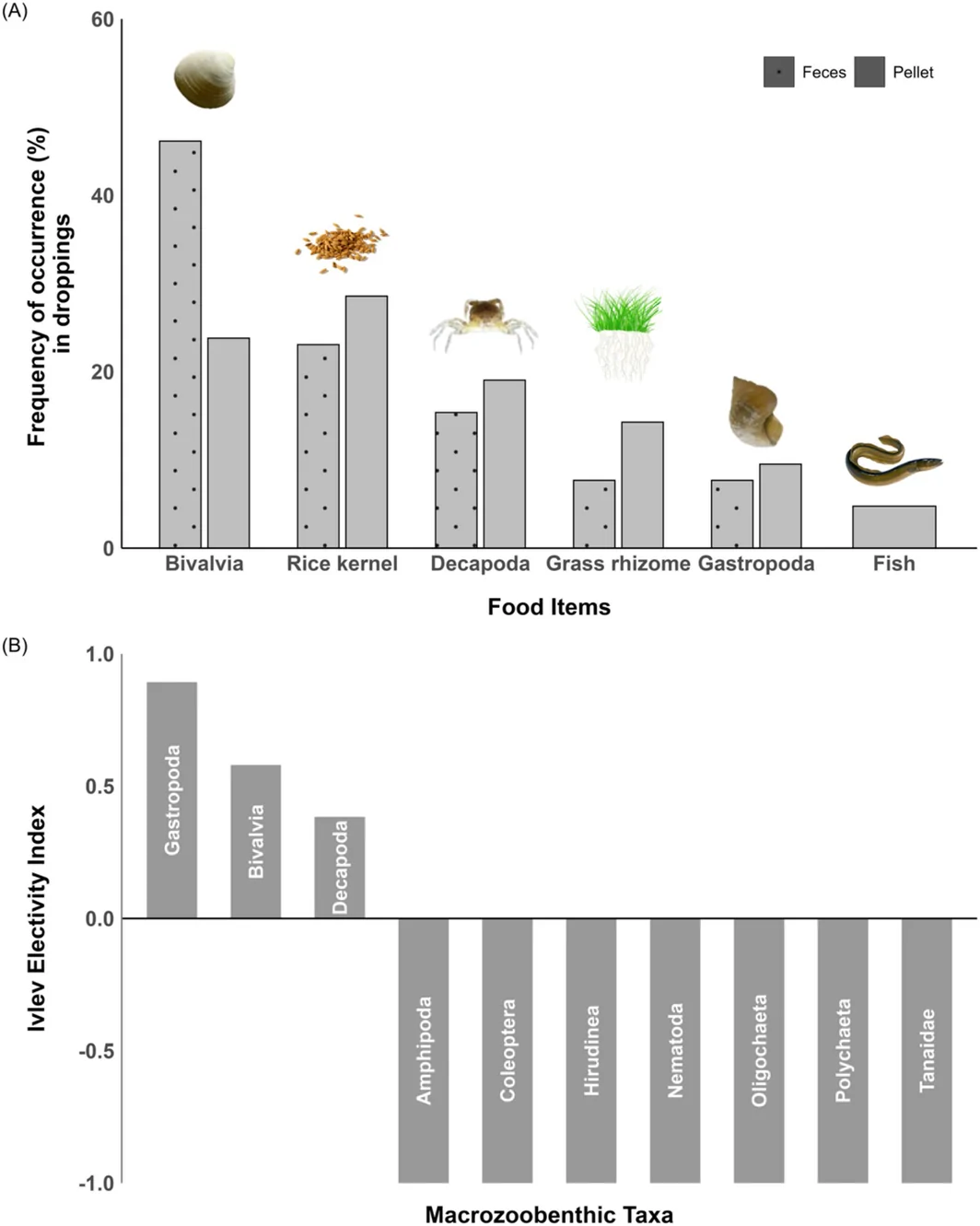
Composition of Black‐tailed Godwits diet with frequency of occurrence (A) based on feces (dotted bar) and pellet (gray bar) analysis. Ivlev's Electivity Index to understand godwits selection of macrozoobenthic taxa from mud samples collected in January 2019 only (B). Positive values of the index indicate preference, negative values indicate avoidance, and values near zero indicate consumption proportional to availability.

## 4 Discussion

Among the foraging areas of godwits in the intertidal mudflats of Nijhum Dweep, 10 microbenthic taxa constitute the benthic community. Five of these taxa are known prey of godwits and dominated the community in terms of abundance. Among the prey taxa, polychaetes and oligochaetes were the most abundant, bivalves and decapods were moderately abundant, and gastropods were the least abundant. Across months, the benthic community composition was broadly similar, with some temporal variation in the relative abundance of bivalves, decapods (increasing), and gastropods (decreasing). Our analysis of droppings revealed that godwits eat primarily bivalves, decapods, and gastropods from the 10 macrozoobenthic taxa available in their intertidal foraging areas. Godwits showed a particularly strong preference for the moderately abundant bivalves, as well as the least abundant gastropods. Besides animal food items, we also present evidence of godwits eating plant items such as rice kernels and rhizomes of Uri Grass—plants that are widely available in the landscape.

Non‐breeding godwits are known to take a variety of animal diets across regions. For example, they have been reported to eat chironomid larvae, ragworm (polychaetes), mud snails (gastropod), brine fly larvae in Spain (Estrella and Masero 2010), bivalves and polychaetes in England (Gill et al. 2001), Portugal (Moreira 1994), and polychaetes, arthropods, bivalves and gastropods in France (Robin et al. 2013, 2015). Our results indicate godwits in the delta prefer benthic prey of hard‐shelled gastropods, bivalves, decapods above soft bodied polychaetes and oligochaetes. Despite the high abundance of polychaetes and oligochaetes in the mud, we did not find their remains in droppings. We rule out missing these soft bodied benthic preys as we found polychaete remains in the droppings of Eurasian Curlew *Numenius arquata* (Ali 2021) using the exact method from the park. Yet, we cannot fully omit the possible consumption of polychaetes because Sultana et al. (2024) made visual observation of godwits taking polychaete worms from the same study area. Because our diet samples were limited to a single location in the month of January, we cannot yet detect spatial and temporal variation in prey use in the Delta. It is possible that some individuals can selectively feed on polychaetes (Gill et al. 2001) and that they can switch to different prey in different time based on the availability and abundance. Also, not all prey individuals are ingestible or profitable because some are oversized or underweight, while others are buried too deeply in the sediment or too costly due to excessive handling times (Piersma et al. 1993). So, it is also possible that hard‐shelled preys are harvestable, that is, accessible, ingestible and profitable (*sensu* Zwarts and Wanink 1993) in the delta in comparison to other available benthic taxa. Beside macrozoobenthic taxa, we found fish remains in a single pellet. We consider this is likely due to opportunistic scavenging, because eel goby *Odontamblyopus rubicundus* , kuchia *Monopterus cuchia* and mudskippers *Periophthalmus* spp. are rife in the mudflats of the delta.

In addition to animal diet, our finding of godwits consuming plant material adds to previous evidence of godwits' herbivory in the East‐Atlantic flyway. There, godwits are known to consume rhizomes of seagrass *Zostera noltei* in France (Robin et al. 2013), and rice kernels in Senegal (Tréca 1994) and Portugal (Lourenço and Piersma 2008). Remarkably, we hardly observed them foraging in rice fields during daytime surveys in Bangladesh. Presence of rice kernels in almost one quarter of dropping samples indicates regular rice consumption in the delta as well, maybe at night and at roost sites in rice fields. Rice cultivation in the park is a regular practice, begins in mid‐June and harvests are reaped around mid‐October which leaves rice kernels on the ground. Future studies using tracking data collected for godwits in Nijhum Dweep between 2021 and 2023 (Das et al. 2025) will allow for a robust assessment of godwit space and habitat use in the delta.

Overall, we show that godwits in the delta feed on a variety of both animal and plant foods. Future studies using larger numbers of droppings across the season and modern techniques, such as DNA metabarcoding, would be useful for detailed understanding of their diet and should focus on diet and niche differences of three co‐occurring subspecies in the delta (Das et al. 2025). Among the diets of godwits in the delta, bivalves and decapods are also consumed by people of the delta and have commercial value. Therefore, we suggest monitoring their stock in the delta as they are under pressure from consumption, commercial use, and habitat alteration. Maintaining a healthy stock of bivalves, decapods, and their habitats in the delta will ensure access to food for both local people and the migratory Asian godwit populations.

## Author Contributions

**Delip K. Das:** conceptualization (lead), data curation (supporting), formal analysis (lead), funding acquisition (lead), investigation (lead), methodology (lead), project administration (lead), resources (equal), software (equal), supervision (supporting), validation (lead), visualization (lead), writing – original draft (lead), writing – review and editing (lead). **Naim Khandakar:** conceptualization (supporting), data curation (lead), formal analysis (lead), investigation (lead), methodology (supporting), project administration (supporting), resources (equal), software (equal), validation (supporting), visualization (lead), writing – original draft (lead), writing – review and editing (equal). **Md. Soab Ali:** data curation (supporting), investigation (supporting), methodology (supporting), writing – review and editing (supporting). **Wouter M. G. Vansteelant:** supervision (lead), validation (lead), writing – review and editing (equal). **El‐Hacen M. El‐Hacen:** supervision (supporting), writing – review and editing (supporting). **Theunis Piersma:** conceptualization (supporting), resources (supporting), supervision (lead), validation (equal), visualization (supporting), writing – original draft (supporting), writing – review and editing (equal).

## Funding

This work was supported by International Wader Study Group Small Grant, Jagannath University Research Grant, and RSPB Small Grant.

## Conflicts of Interest

The authors declare no conflicts of interest.

## Supporting information


**Table S1:** Date and location of the collected droppings and mud samples.
**Table S2:** Results of SIMPER analysis comparing macrozoobenthic taxa among sampling months. “average” indicates the average contribution of each taxon to Bray–Curtis dissimilarity between months, and “SD” represents the standard deviation of that contribution. “ratio” gives the ratio of average contribution to its standard deviation (average/SD). “ava” and “avb” denote the average abundance of each taxon in the first and second month of each contrast, respectively. “cumsum” indicates the cumulative proportional contribution of taxa to total dissimilarity. “*p*” represents permutation‐based *p*‐values for taxon contributions.

## Data Availability

The data supporting the findings of this study are available in Dryad at https://doi.org/10.5061/dryad.3tx95x6z0.

## References

[ece374479-bib-0001] Ali, S. 2021. “Population Status and Non‐Breeding Ecology of Eurasian Curlew, Great Knot and Curlew Sandpiper at Nijhum Dweep National Park, Bangladesh.” MSc. Thesis, Jagannath University, Bangladesh.

[ece374479-bib-0002] Alves, J. A. , T. G. Gunnarsson , P. M. Potts , W. J. Sutherland , and J. A. Gill . 2013. “Sex‐Biases in Distribution and Resource Use at Different Spatial Scales in a Migratory Shorebird.” Ecology and Evolution 3: 1079–1090. 10.1002/ece3.503 PMC3631415 23610645

[ece374479-bib-0003] BirdLife International . 2026. “Species Factsheet: *Limosa limosa.”* BirdLife Data Zone. http://datazone.birdlife.org/species/factsheet/black‐tailed‐godwit‐limosa‐limosa.

[ece374479-bib-0004] Brammer, H. 2014. “Bangladesh's Dynamic Coastal Regions and Sea‐Level Rise.” Climate Risk Management 1: 51–62.

[ece374479-bib-0005] Catry, T. , J. A. Alves , J. A. Gill , T. G. Gunnarsson , and J. P. Granadeiro . 2012. “Sex Promotes Spatial and Dietary Segregation in a Migratory Shorebird During the Non‐Breeding Season.” PLoS One 7: e33811. 10.1371/journal.pone.0033811 PMC3316497 22479448

[ece374479-bib-0006] Chan, Y.‐C. , D. T. C. Chan , T. L. Tibbitts , C. J. Hassell , and T. Piersma . 2023. “Site Fidelity of Migratory Shorebirds Facing Habitat Deterioration: Insights From Satellite Tracking and Mark‐Resighting.” Movement Ecology 11: 79. 10.1186/s40462-023-00443-9 PMC10740345 38129912

[ece374479-bib-0007] Correia, E. , J. Granadeiro , B. Santos , A. Regalla , V. Mata , and T. Catry . 2023. “Trophic Ecology of a Migratory Shorebird Community at a Globally Important Non‐Breeding Site: Combining DNA Metabarcoding and Conventional Techniques.” Marine Ecology Progress Series 705: 127–144.

[ece374479-bib-0008] Das, D. K. , N. Khandakar , I. Sultana , et al. 2022. “Site Use by Non‐Breeding Black‐Tailed Godwits at Nijhum Dweep National Park, Bangladesh.” Wader Study 129: 14–21.

[ece374479-bib-0009] Das, D. K. , W. M. Vansteelant , B. Zhu , et al. 2025. “Three Subspecies of Black‐Tailed Godwit Share Non‐Breeding Sites in the World's Largest River Delta.” Avian Research 16: 100226.

[ece374479-bib-0010] Emran, A. , M. A. Rob , M. H. Kabir , and M. N. Islam . 2015. “Modeling Spatio‐Temporal Shoreline and Areal Dynamics of Coastal Island Using Geospatial Technique.” Modeling Earth Systems and Environment 2: 4.

[ece374479-bib-0011] Estrella, S. M. , and J. A. Masero . 2010. “Prey and Prey Size Selection by the Near‐Threatened Black‐Tailed Godwit Foraging in Non‐Tidal Areas During Migration.” Waterbirds 33: 293–299.

[ece374479-bib-0012] Gill, J. A. , W. J. Sutherland , and K. Norris . 2001. “Depletion Models Can Predict Shorebird Distribution at Different Spatial Scales.” Proceedings of the Royal Society B 268: 369–376. 10.1098/rspb.2000.1386 PMC1088616 11270433

[ece374479-bib-0015] Green, A. J. , and M. I. Sánchez . 2006. “Passive Internal Dispersal of Insect Larvae by Migratory Birds.” Biology Letters 2: 55–57. 10.1098/rsbl.2005.0413 PMC1617192 17148325

[ece374479-bib-0016] Hayhow, D. B. 2009. “Consequences of Winter Habitat Use in a Migratory Shorebird.” PhD thesis, University of East Anglia, Norwich, UK.

[ece374479-bib-0017] Hossain, K. T. , M. Salauddin , and I. A. Tanim . 2016. “Assessment of the Dynamics of Coastal Island in Bangladesh Using Geospatial Techniques: Domar Char.” Journal of the Asiatic Society of Bangladesh, Science 42: 219–228.

[ece374479-bib-0018] Hossain, M. S. , S. Sharifuzzaman , S. R. Chowdhury , et al. 2014. Sea Shells of Bangladesh: Marine Gastropod and Bivalve Molluscs Biodiversity. Centre for Coast, Climate and Communities & Institute of Marine Sciences and Fisheries, University of Chittagong.

[ece374479-bib-0019] Islam, M. S. 2021. “Assessing the Dynamics of Land Cover and Shoreline Changes of Nijhum Dwip (Island) of Bangladesh Using Remote Sensing and GIS Techniques.” Regional Studies in Marine Science 41: 101578.

[ece374479-bib-0020] Ivlev, V. S. 1961. Experimental Ecology of the Feeding of Fishes. Yale University Press.

[ece374479-bib-0021] Kabir, M. A. , M. Salauddin , K. T. Hossain , I. A. Tanim , M. M. H. Saddam , and A. U. Ahmad . 2020. “Assessing the Shoreline Dynamics of Hatiya Island of Meghna Estuary in Bangladesh Using Multiband Satellite Imageries and Hydro‐Meteorological Data.” Regional Studies in Marine Science 35: 101167.

[ece374479-bib-0022] Kabir, S. M. H. , M. Ahmad , A. T. A. Ahmed , et al. 2008. “Encyclopedia of Flora and Fauna of Bangladesh.” In Platyhelminthes‐Nematoda, vol. 15. Asiatic Society of Bangladesh.

[ece374479-bib-0023] Kabir, S. M. H. , M. Ahmad , A. T. A. Ahmed , et al. 2009. “Encyclopedia of Flora and Fauna of Bangladesh.” In Annelida Echinodermata Acanthocephala and Minor Phyla, vol. 16. Asiatic Society of Bangladesh.

[ece374479-bib-0024] Khandakar, N. , S. Islam , I. Sultana , et al. 2026. “Avifuana of Nijhum Dweep National Park in Bangladesh, an East Asian‐Australasian Flyway Network Site and Important Bird and Biodiversity Area in the World's Largest River Delta.” Journal of the Bombay Natural History Society 123: 3–12.

[ece374479-bib-0025] Lourenço, P. M. , and T. Piersma . 2008. “Stopover Ecology of Black‐Tailed Godwits *Limosa limosa limosa* in Portuguese Rice Fields: A Guide on Where to Feed in Winter.” Bird Study 55: 194–202.

[ece374479-bib-0026] Marra, P. P. , C. E. Studds , S. Wilson , T. S. Sillett , T. W. Sherry , and R. T. Holmes . 2015. “Non‐Breeding Season Habitat Quality Mediates the Strength of Density‐Dependence for a Migratory Bird.” Proceedings of the Royal Society B: Biological Sciences 282: 1811. 10.1098/rspb.2015.0624 PMC4528546 26136445

[ece374479-bib-0027] Moreira, F. 1994. “Diet, Prey‐Size Selection and Intake Rates of Black‐Tailed Godwits *Limosa limosa* Feeding on Mudflats.” Ibis 136: 349–355.

[ece374479-bib-0028] Oksanen, J. , F. G. Blanchet , M. Friendly , et al. 2018. “VEGAN: Community Ecology Package.” R Package v. 2.5‐2. https://cran.r‐project.org/package=vegan.

[ece374479-bib-0029] Peng, H.‐B. 2024. “Exploring Shorebird Conservation From on and in the Mudflats.” PhD Thesis, University of Groningen, Groningen, the Netherlands.

[ece374479-bib-0030] Penning, E. , S. Esroy , L. L. Govers , et al. 2026. “Avian Prey Exhibit Repeatable Abundances on Intertidal Mudflats.” Estuarine, Coastal and Shelf Science 336: 109900.

[ece374479-bib-0031] Penning, E. , Y. I. Verkuil , L. Klunder , and J. Reneerkens . 2022. “Sanderlings Feed on a Diverse Spectrum of Prey Worldwide but Primarily Rely on Brown Shrimp in the Wadden Sea.” Ardea 110, no. 1: 87–220.

[ece374479-bib-0032] Perez‐Hurtado, A. , J. D. Goss‐Custard , and F. Garcia . 1997. “The Diet of Wintering Waders in Cádiz Bay, Southwest Spain.” Bird Study 44: 45–52.

[ece374479-bib-0034] Piersma, T. , P. de Goeij , and I. Tulp . 1993. “An Evaluation of Intertidal Feeding Habitats From a Shorebird Perspective: Towards Relevant Comparisons Between Temperate and Tropical Mudflats.” Netherlands Journal of Sea Research 31: 503–512.

[ece374479-bib-0035] Piersma, T. , R. E. Gill Jr. , D. R. Ruthrauff , C. G. Guglielmo , J. R. Conklin , and C. M. Handel . 2022. “The Pacific as the World's Greatest Theater of Bird Migration: Extreme Flights Spark Questions About Physiological Capabilities, Behavior, and the Evolution of Migratory Pathways.” Ornithology 139: 1–29.

[ece374479-bib-0036] Rahman, S. , M. S. Islam , M. N. H. Khan , and M. Touhiduzzaman . 2019. “Climate Change Adaptation and Disaster Risk Reduction (DRR) Through Coastal Afforestation in South‐Central Coast of Bangladesh.” Management of Environmental Quality: An International Journal 30: 498–517.

[ece374479-bib-0037] Robin, F. , F. Meunier , F. Corre , et al. 2015. “Site and Prey Selection by Wintering Black‐Tailed Godwit *Limosa limosa islandica* Feeding on Seagrass Beds and Bare Mudflats on the Central Atlantic Coast of France.” Revue D'écologie (La Terre et La Vie) 70: 134–147.

[ece374479-bib-0038] Robin, F. , T. Piersma , F. Meunier , and P. Bocher . 2013. “Expansion Into an Herbivorous Niche by a Customary Carnivore: Black‐Tailed Godwits Feeding on Rhizomes of *Zostera* at a Newly Established Wintering Site.” Condor 115: 340–347.

[ece374479-bib-0039] Sánchez, M. I. , A. J. Green , and E. M. Castellanos . 2006. “Temporal and Spatial Variation of an Aquatic Invertebrate Community Subjected to Avian Predation at the Odiel Salt Pans (SW Spain).” Archiv für Hydrobiologie 166: 199–223.

[ece374479-bib-0040] Siddiqui, K. U. , M. A. Islam , S. M. H. Kabir , et al. 2007. Encyclopedia of Flora and Fauna of Bangladesh, Vol. 17: Molluscs. Asiatic Society of Bangladesh.

[ece374479-bib-0041] Studds, C. E. , B. E. Kendall , N. J. Murray , et al. 2017. “Rapid Population Decline in Migratory Shorebirds Relying on Yellow Sea Tidal Mudflats as Stopover Sites.” Nature Communications 8: 14895. 10.1038/ncomms14895 PMC5399291 28406155

[ece374479-bib-0042] Sultana, I. , N. Khandakar , D. K. Das , S. Qader , and A. Viswanathan . 2024. “Polychaete Worm Density Correlates With Aggregate Shorebird Density, Prey Intake Rate and Foraging Success at an Intertidal Mudflat in Bangladesh.” Ardea 112: 113–130.

[ece374479-bib-0043] Tréca, B. 1994. “The Diet of Ruffs and Black‐Tailed Godwits in Senegal.” Ostrich 65: 256–263.

[ece374479-bib-0044] van de Kam, J. , B. J. Ens , T. Piersma , and L. Zwarts . 2004. Shorebirds. An Illustrated Behavioural Ecology. KNNV Publishers.

[ece374479-bib-0045] Wickham, H. 2016. ggplot2: Elegant Graphics for Data Analysis. Springer‐Verlag. https://ggplot2.tidyverse.org.

[ece374479-bib-0046] Winkler, D. W. , J. Shamoun‐Baranes , and T. Piersma . 2016. “Chapter 12: Avian Migration and Dispersal.” In Cornell Lab of Ornithology Handbook of Bird Biology, edited by I. J. Lovette and J. W. Fitzpatrick , 3rd ed., 452–492. Wiley.

[ece374479-bib-0047] Zhu, B. R. , C. J. Hassell , Y. I. Verkuil , et al. 2020. “Size, Shape and Sex Differences in Three Subspecies of Black‐Tailed Godwits *Limosa limosa* .” Bird Study 67: 45–62.

[ece374479-bib-0048] Zhu, B. R. , M. A. Verhoeven , T. B. Craft , et al. 2026. “Male and Female Black‐Tailed Godwits * Limosa limosa Bohaii* Show Differences in Habitat Use and Daily Rhythms During Spring Staging in China.” Journal of Ornithology 167: 999–1008.

[ece374479-bib-0049] Zhu, B. R. , M. A. Verhoeven , C. J. Hassell , et al. 2023. “Predicting the Non‐Breeding Distributions of the Two Asian Subspecies of Black‐Tailed Godwit Using Morphological Information.” Avian Research 14: 100069.

[ece374479-bib-0050] Zhu, B. R. , Y. I. Verkuil , J. R. Conklin , et al. 2021. “Discovery of a Morphologically and Genetically Distinct Population of Black‐Tailed Godwits in the East Asian‐Australasian Flyway.” Ibis 163: 448–462.

[ece374479-bib-0051] Zwarts, L. , and J. H. Wanink . 1993. “How the Food Supply Harvestable by Waders in the Wadden Sea Depends on the Variation in Energy Density, Body Weight, Biomass, Burying Depth and Behaviour of Tidal‐Flat Invertebrates.” Netherlands Journal of Sea Research 31: 441–476.

