# Supplementary material for: Black‐Tailed Godwits Eat Animal and Plant Material at a Key Non‐Breeding Site at the Overlap of Two Asian Flyways
Source: Ecol Evol. 2026 Oct 18;16(10):e74479. doi: 10.1002/ece3.74479 (PMC13677280; doi:10.1002/ece3.74479)
Supplement: Supplementary file 1 [file ECE3-16-e74479-s001.docx]

Table S1: Date and location of the collected droppings and mud samples.

| **Date** | **Sample type** | **Number** | **Latitude (°N)** | **Longitude (°E)** |
| --- | --- | --- | --- | --- |
| 15 January 2019 | Pellet | 15 | 22.055278 | 91.052778 |
| 15 January 2019 | Feces | 15 | 22.055278 | 91.052778 |
| 25 October 2018 | Mud sample | 5 | 22.073047 | 91.055942 |
| 25 October 2018 | Mud sample | 5 | 22.071728 | 91.055447 |
| 25 October 2018 | Mud sample | 5 | 22.070187 | 91.055641 |
| 25 October 2018 | Mud sample | 5 | 22.069986 | 91.055871 |
| 25 October 2018 | Mud sample | 5 | 22.069839 | 91.055394 |
| 25 October 2018 | Mud sample | 5 | 22.069842 | 91.055233 |
| 24 November 2018 | Mud sample | 5 | 22.074133 | 91.096944 |
| 24 November 2018 | Mud sample | 5 | 22.074322 | 91.097222 |
| 24 November 2018 | Mud sample | 5 | 22.074383 | 91.097222 |
| 24 November 2018 | Mud sample | 5 | 22.074247 | 91.097777 |
| 24 November 2018 | Mud sample | 5 | 22.074497 | 91.097777 |
| 24 November 2018 | Mud sample | 5 | 22.074736 | 91.098055 |
| 24 November 2018 | Mud sample | 5 | 22.074575 | 91.096944 |
| 24 November 2018 | Mud sample | 5 | 22.055661 | 91.103333 |
| 24 November 2018 | Mud sample | 5 | 22.054961 | 91.103611 |
| 24 November 2018 | Mud sample | 5 | 22.054925 | 91.103611 |
| 24 November 2018 | Mud sample | 5 | 22.054825 | 91.103611 |
| 17 December 2018 | Mud sample | 5 | 22.040422 | 91.027847 |
| 17 December 2018 | Mud sample | 5 | 22.039428 | 91.026172 |
| 18 December 2018 | Mud sample | 5 | 22.038875 | 90.973792 |
| 18 December 2018 | Mud sample | 5 | 22.038555 | 90.973455 |
| 18 December 2018 | Mud sample | 5 | 22.038293 | 90.973134 |
| 18 December 2018 | Mud sample | 5 | 22.037976 | 90.972760 |
| 18 December 2018 | Mud sample | 5 | 22.037734 | 90.972419 |
| 25 December 2018 | Mud sample | 5 | 22.109465 | 91.138201 |
| 25 December 2018 | Mud sample | 5 | 22.109568 | 91.138206 |
| 25 December 2018 | Mud sample | 5 | 22.109779 | 91.138213 |
| 25 December 2018 | Mud sample | 5 | 22.109930 | 91.138272 |
| 25 December 2018 | Mud sample | 5 | 22.110059 | 91.138356 |
| 25 December 2018 | Mud sample | 5 | 22.110188 | 91.138434 |
| 15 January 2019 | Mud sample | 5 | 22.040200 | 91.047737 |
| 15 January 2019 | Mud sample | 5 | 22.040533 | 91.046007 |
| 15 January 2019 | Mud sample | 5 | 22.040230 | 91.045878 |
| 15 January 2019 | Mud sample | 5 | 22.039969 | 91.045798 |
| 15 January 2019 | Mud sample | 5 | 22.039776 | 91.045788 |
| 15 January 2019 | Mud sample | 5 | 22.039570 | 91.045762 |
| 15 January 2019 | Mud sample | 5 | 22.062195 | 91.048973 |
| 15 January 2019 | Mud sample | 5 | 22.061982 | 91.048924 |
| 15 January 2019 | Mud sample | 5 | 22.061818 | 91.048854 |
| 15 January 2019 | Mud sample | 5 | 22.061538 | 91.048835 |
| 15 January 2019 | Mud sample | 5 | 22.061291 | 91.048787 |
| 15 January 2019 | Mud sample | 5 | 22.061034 | 91.048738 |
| 15 January 2019 | Mud sample | 5 | 22.060871 | 91.048733 |
| 15 January 2019 | Mud sample | 5 | 22.060710 | 91.048668 |
| 15 January 2019 | Mud sample | 5 | 22.060547 | 91.048541 |
| 15 January 2019 | Mud sample | 5 | 22.060506 | 91.048284 |
| 15 January 2019 | Mud sample | 5 | 22.060668 | 91.048109 |
| 15 January 2019 | Mud sample | 5 | 22.060406 | 91.048441 |
| 15 January 2019 | Mud sample | 5 | 22.059904 | 91.048341 |
| 17 January 2019 | Mud sample | 5 | 22.093216 | 91.112777 |
| 17 January 2019 | Mud sample | 5 | 22.094644 | 91.112777 |
| 17 January 2019 | Mud sample | 5 | 22.095327 | 91.112777 |
| 17 January 2019 | Mud sample | 5 | 22.097302 | 91.111944 |
| 17 January 2019 | Mud sample | 5 | 22.097844 | 91.113055 |
| 17 January 2019 | Mud sample | 5 | 22.109583 | 91.137717 |
| 17 January 2019 | Mud sample | 5 | 22.109767 | 91.137884 |
| 17 January 2019 | Mud sample | 5 | 22.109795 | 91.138114 |
| 17 January 2019 | Mud sample | 5 | 22.109830 | 91.138408 |
| 17 January 2019 | Mud sample | 5 | 22.109894 | 91.138540 |
| 17 January 2019 | Mud sample | 5 | 22.109969 | 91.138695 |
| 17 January 2019 | Mud sample | 5 | 22.109889 | 91.136427 |
| 17 January 2019 | Mud sample | 5 | 22.110166 | 91.136195 |
| 17 January 2019 | Mud sample | 5 | 22.110376 | 91.135721 |
| 17 January 2019 | Mud sample | 5 | 22.110505 | 91.135211 |
| 17 January 2019 | Mud sample | 5 | 22.110582 | 91.134916 |
| 15 February 2019 | Mud sample | 5 | 22.037529 | 90.971971 |
| 15 February 2019 | Mud sample | 5 | 22.041230 | 90.971183 |
| 15 February 2019 | Mud sample | 5 | 22.041283 | 90.971104 |
| 15 February 2019 | Mud sample | 5 | 22.040268 | 90.970712 |
| 15 February 2019 | Mud sample | 5 | 22.040000 | 90.970643 |
| 15 February 2019 | Mud sample | 5 | 22.039735 | 90.970452 |
| 15 February 2019 | Mud sample | 5 | 22.039449 | 90.970359 |
| 15 February 2019 | Mud sample | 5 | 22.039081 | 90.970454 |
| 15 February 2019 | Mud sample | 5 | 22.037877 | 90.971067 |
| 18 February 2019 | Mud sample | 5 | 22.073110 | 91.096723 |
| 18 February 2019 | Mud sample | 5 | 22.073122 | 91.096907 |
| 18 February 2019 | Mud sample | 5 | 22.073116 | 91.097161 |
| 18 February 2019 | Mud sample | 5 | 22.073197 | 91.097263 |
| 18 February 2019 | Mud sample | 5 | 22.073218 | 91.097449 |
| 18 February 2019 | Mud sample | 5 | 22.073258 | 91.097599 |
| 18 February 2019 | Mud sample | 5 | 22.073423 | 91.097559 |
| 18 February 2019 | Mud sample | 5 | 22.073442 | 91.097348 |
| 18 February 2019 | Mud sample | 5 | 22.073462 | 91.097178 |
| 18 February 2019 | Mud sample | 5 | 22.073492 | 91.097053 |
| 18 February 2019 | Mud sample | 5 | 22.073508 | 91.096872 |
| 29 March 2019 | Mud sample | 5 | 22.059594 | 91.048318 |
| 29 March 2019 | Mud sample | 5 | 22.061926 | 91.048523 |
| 29 March 2019 | Mud sample | 5 | 22.061849 | 91.048384 |
| 29 March 2019 | Mud sample | 5 | 22.061769 | 91.048160 |
| 29 March 2019 | Mud sample | 5 | 22.061641 | 91.048119 |
| 30 March 2019 | Mud sample | 5 | 22.033609 | 90.976716 |
| 30 March 2019 | Mud sample | 5 | 22.033380 | 90.976615 |
| 30 March 2019 | Mud sample | 5 | 22.033227 | 90.976428 |
| 30 March 2019 | Mud sample | 5 | 22.033046 | 90.976320 |
| 30 March 2019 | Mud sample | 5 | 22.032856 | 90.976178 |
| 30 March 2019 | Mud sample | 5 | 22.032557 | 90.976017 |
| 30 March 2019 | Mud sample | 5 | 22.032251 | 90.975858 |
| 30 March 2019 | Mud sample | 5 | 22.032370 | 90.975836 |
| 30 March 2019 | Mud sample | 5 | 22.032490 | 90.975672 |
| 30 March 2019 | Mud sample | 5 | 22.032614 | 90.975529 |
| 30 March 2019 | Mud sample | 5 | 22.032736 | 90.975397 |
| 30 March 2019 | Mud sample | 5 | 22.032185 | 90.975945 |
| 30 March 2019 | Mud sample | 5 | 22.032008 | 90.976036 |
| 30 March 2019 | Mud sample | 5 | 22.031826 | 90.976056 |
| 31 March 2019 | Mud sample | 5 | 22.103919 | 91.137575 |
| 31 March 2019 | Mud sample | 5 | 22.104111 | 91.137716 |
| 31 March 2019 | Mud sample | 5 | 22.104388 | 91.137857 |
| 31 March 2019 | Mud sample | 5 | 22.104500 | 91.137979 |
| 31 March 2019 | Mud sample | 5 | 22.104925 | 91.138272 |
| 31 March 2019 | Mud sample | 5 | 22.105070 | 91.138356 |
| 31 March 2019 | Mud sample | 5 | 22.105188 | 91.138579 |
| 31 March 2019 | Mud sample | 5 | 22.105429 | 91.138657 |
| 02 April 2019 | Mud sample | 5 | 22.073113 | 91.096788 |
| 02 April 2019 | Mud sample | 5 | 22.073189 | 91.096914 |
| 02 April 2019 | Mud sample | 5 | 22.073310 | 91.097044 |
| 02 April 2019 | Mud sample | 5 | 22.073477 | 91.097230 |
| 02 April 2019 | Mud sample | 5 | 22.073563 | 91.097391 |
| 02 April 2019 | Mud sample | 5 | 22.073467 | 91.097524 |
| 02 April 2019 | Mud sample | 5 | 22.073424 | 91.097627 |
| 02 April 2019 | Mud sample | 5 | 22.073275 | 91.097376 |

Table S2: Results of SIMPER analysis comparing macrozoobenthic taxa among sampling months. “average” indicates the average contribution of each taxon to Bray–Curtis dissimilarity between months, and “SD” represents the standard deviation of that contribution. “ratio” gives the ratio of average contribution to its standard deviation (average/SD). “ava” and “avb” denote the average abundance of each taxon in the first and second month of each contrast, respectively. “cumsum” indicates the cumulative proportional contribution of taxa to total dissimilarity. “*p*” represents permutation-based *p*-values for taxon contributions.

Contrast: October 2018–November 2018

| Taxa | average | SD | ratio | ava | avb | cumsum | *p* |
| --- | --- | --- | --- | --- | --- | --- | --- |
| Polychaeta | 0.1170 | 0.1009 | 1.1592 | 0.4372 | 0.4834 | 0.2039 | 0.06 |
| Oligochaeta | 0.1090 | 0.1012 | 1.0773 | 0.4518 | 0.4734 | 0.3939 | 0.391 |
| Bivalvia | 0.0787 | 0.0912 | 0.8634 | 0.1755 | 0.1758 | 0.5311 | 0.998 |
| Gastropoda | 0.0685 | 0.0829 | 0.8263 | 0.1611 | 0.1457 | 0.6505 | 0.001 |
| Nematoda | 0.0593 | 0.0797 | 0.7443 | 0.1257 | 0.1263 | 0.7540 | 0.001 |
| Decapoda | 0.0531 | 0.0715 | 0.7423 | 0.0000 | 0.1872 | 0.8465 | 1 |
| Hirudinea | 0.0334 | 0.0629 | 0.5312 | 0.0597 | 0.0718 | 0.9047 | 0.009 |
| Tanaidae | 0.0244 | 0.0527 | 0.4628 | 0.0308 | 0.0638 | 0.9472 | 0.01 |
| Amphipoda | 0.0229 | 0.0557 | 0.4111 | 0.0571 | 0.0276 | 0.9871 | 0.959 |
| Coleoptera | 0.0074 | 0.0301 | 0.2456 | 0.0000 | 0.0268 | 1.0000 | 0.336 |

Contrast: October 2018–December 2018

| Taxa | average | SD | ratio | ava | avb | cumsum | *p* |
| --- | --- | --- | --- | --- | --- | --- | --- |
| Polychaeta | 0.1257 | 0.1130 | 1.1123 | 0.4372 | 0.4220 | 0.2204 | 0.003 |
| Oligochaeta | 0.1255 | 0.1146 | 1.0949 | 0.4518 | 0.5705 | 0.4405 | 0.014 |
| Bivalvia | 0.0879 | 0.0984 | 0.8932 | 0.1755 | 0.2075 | 0.5947 | 0.969 |
| Gastropoda | 0.0602 | 0.0866 | 0.6949 | 0.1611 | 0.0674 | 0.7003 | 0.001 |
| Decapoda | 0.0544 | 0.0734 | 0.7412 | 0.0000 | 0.1911 | 0.7957 | 1 |
| Nematoda | 0.0474 | 0.0808 | 0.5860 | 0.1257 | 0.0462 | 0.8788 | 0.001 |
| Hirudinea | 0.0291 | 0.0674 | 0.4321 | 0.0597 | 0.0420 | 0.9298 | 0.028 |
| Amphipoda | 0.0286 | 0.0604 | 0.4732 | 0.0571 | 0.0487 | 0.9799 | 0.858 |
| Tanaidae | 0.0102 | 0.0342 | 0.2970 | 0.0308 | 0.0059 | 0.9978 | 0.545 |
| Coleoptera | 0.0013 | 0.0102 | 0.1257 | 0.0000 | 0.0055 | 1.0000 | 0.827 |

Contrast: October 2018–January 2019

| Taxa | average | SD | ratio | ava | avb | cumsum | *p* |
| --- | --- | --- | --- | --- | --- | --- | --- |
| Polychaeta | 0.1226 | 0.1070 | 1.1456 | 0.4372 | 0.5378 | 0.2118 | 0.007 |
| Oligochaeta | 0.1192 | 0.1063 | 1.1215 | 0.4518 | 0.4102 | 0.4177 | 0.045 |
| Bivalvia | 0.0984 | 0.1020 | 0.9652 | 0.1755 | 0.2555 | 0.5876 | 0.591 |
| Decapoda | 0.0680 | 0.0972 | 0.7001 | 0.0000 | 0.2175 | 0.7051 | 0.999 |
| Gastropoda | 0.0526 | 0.0856 | 0.6148 | 0.1611 | 0.0133 | 0.7960 | 0.002 |
| Nematoda | 0.0431 | 0.0813 | 0.5302 | 0.1257 | 0.0175 | 0.8704 | 0.003 |
| Amphipoda | 0.0408 | 0.0717 | 0.5696 | 0.0571 | 0.0988 | 0.9409 | 0.312 |
| Hirudinea | 0.0203 | 0.0560 | 0.3619 | 0.0597 | 0.0086 | 0.9759 | 0.171 |
| Tanaidae | 0.0111 | 0.0370 | 0.3009 | 0.0308 | 0.0087 | 0.9951 | 0.397 |
| Coleoptera | 0.0028 | 0.0264 | 0.1079 | 0.0000 | 0.0082 | 1.0000 | 0.753 |

Contrast: October 2018–February 2019

| Taxa | average | SD | ratio | ava | avb | cumsum | *p* |
| --- | --- | --- | --- | --- | --- | --- | --- |
| Polychaeta | 0.1087 | 0.0874 | 1.2436 | 0.4372 | 0.4262 | 0.1837 | 0.249 |
| Bivalvia | 0.0988 | 0.0855 | 1.1560 | 0.1755 | 0.3144 | 0.3508 | 0.51 |
| Oligochaeta | 0.0979 | 0.0840 | 1.1654 | 0.4518 | 0.4317 | 0.5164 | 0.831 |
| Decapoda | 0.0873 | 0.0907 | 0.9627 | 0.0000 | 0.2926 | 0.6640 | 0.631 |
| Gastropoda | 0.0594 | 0.0809 | 0.7341 | 0.1611 | 0.0883 | 0.7644 | 0.002 |
| Nematoda | 0.0560 | 0.0784 | 0.7146 | 0.1257 | 0.1089 | 0.8591 | 0.001 |
| Amphipoda | 0.0284 | 0.0586 | 0.4850 | 0.0571 | 0.0538 | 0.9072 | 0.893 |
| Hirudinea | 0.0278 | 0.0588 | 0.4720 | 0.0597 | 0.0469 | 0.9541 | 0.03 |
| Tanaidae | 0.0228 | 0.0494 | 0.4618 | 0.0308 | 0.0579 | 0.9926 | 0.012 |
| Coleoptera | 0.0044 | 0.0247 | 0.1762 | 0.0000 | 0.0152 | 1.0000 | 0.565 |

Contrast: October 2018–March 2019

| Taxa | average | SD | ratio | ava | avb | cumsum | *p* |
| --- | --- | --- | --- | --- | --- | --- | --- |
| Polychaeta | 0.1264 | 0.1087 | 1.1628 | 0.4372 | 0.4949 | 0.2092 | 0.001 |
| Oligochaeta | 0.1205 | 0.1053 | 1.1437 | 0.4518 | 0.3671 | 0.4087 | 0.035 |
| Bivalvia | 0.1165 | 0.1056 | 1.1026 | 0.1755 | 0.3501 | 0.6015 | 0.003 |
| Decapoda | 0.0766 | 0.0995 | 0.7700 | 0.0000 | 0.2443 | 0.7283 | 0.976 |
| Gastropoda | 0.0505 | 0.0845 | 0.5971 | 0.1611 | 0.0000 | 0.8119 | 0.002 |
| Amphipoda | 0.0409 | 0.0726 | 0.5626 | 0.0571 | 0.0976 | 0.8795 | 0.355 |
| Nematoda | 0.0400 | 0.0803 | 0.4983 | 0.1257 | 0.0000 | 0.9458 | 0.01 |
| Hirudinea | 0.0217 | 0.0576 | 0.3769 | 0.0597 | 0.0144 | 0.9817 | 0.133 |
| Tanaidae | 0.0088 | 0.0323 | 0.2710 | 0.0308 | 0.0000 | 0.9962 | 0.634 |
| Coleoptera | 0.0023 | 0.0221 | 0.1046 | 0.0000 | 0.0066 | 1.0000 | 0.805 |

Contrast: November 2018–December 2018

| Taxa | average | SD | ratio | ava | avb | cumsum | *p* |
| --- | --- | --- | --- | --- | --- | --- | --- |
| Oligochaeta | 0.1061 | 0.1031 | 1.0295 | 0.4734 | 0.5705 | 0.2040 | 0.495 |
| Polychaeta | 0.1022 | 0.1018 | 1.0039 | 0.4834 | 0.4220 | 0.4004 | 0.513 |
| Bivalvia | 0.0748 | 0.0796 | 0.9401 | 0.1758 | 0.2075 | 0.5442 | 1 |
| Decapoda | 0.0715 | 0.0750 | 0.9527 | 0.1872 | 0.1911 | 0.6815 | 0.994 |
| Gastropoda | 0.0497 | 0.0704 | 0.7057 | 0.1457 | 0.0674 | 0.7770 | 0.001 |
| Nematoda | 0.0407 | 0.0609 | 0.6673 | 0.1263 | 0.0462 | 0.8551 | 0.001 |
| Hirudinea | 0.0291 | 0.0597 | 0.4870 | 0.0718 | 0.0420 | 0.9110 | 0.004 |
| Amphipoda | 0.0192 | 0.0466 | 0.4122 | 0.0276 | 0.0487 | 0.9480 | 0.999 |
| Tanaidae | 0.0188 | 0.0476 | 0.3942 | 0.0638 | 0.0059 | 0.9840 | 0.046 |
| Coleoptera | 0.0083 | 0.0307 | 0.2711 | 0.0268 | 0.0055 | 1.0000 | 0.217 |

Contrast: November 2018–January 2019

| Taxa | average | SD | ratio | ava | avb | cumsum | *p* |
| --- | --- | --- | --- | --- | --- | --- | --- |
| Oligochaeta | 0.1035 | 0.0978 | 1.0581 | 0.4734 | 0.4102 | 0.1960 | 0.687 |
| Polychaeta | 0.0949 | 0.0925 | 1.0267 | 0.4834 | 0.5378 | 0.3757 | 0.9 |
| Bivalvia | 0.0854 | 0.0866 | 0.9866 | 0.1758 | 0.2555 | 0.5374 | 1 |
| Decapoda | 0.0817 | 0.0864 | 0.9450 | 0.1872 | 0.2175 | 0.6921 | 0.946 |
| Gastropoda | 0.0427 | 0.0675 | 0.6317 | 0.1457 | 0.0133 | 0.7729 | 0.002 |
| Nematoda | 0.0374 | 0.0608 | 0.6140 | 0.1263 | 0.0175 | 0.8436 | 0.004 |
| Amphipoda | 0.0320 | 0.0618 | 0.5181 | 0.0276 | 0.0988 | 0.9042 | 0.871 |
| Hirudinea | 0.0213 | 0.0489 | 0.4360 | 0.0718 | 0.0086 | 0.9446 | 0.096 |
| Tanaidae | 0.0195 | 0.0489 | 0.3998 | 0.0638 | 0.0087 | 0.9816 | 0.012 |
| Coleoptera | 0.0097 | 0.0375 | 0.2589 | 0.0268 | 0.0082 | 1.0000 | 0.102 |

Contrast: November 2018–February 2019

| Taxa | average | SD | ratio | ava | avb | cumsum | *p* |
| --- | --- | --- | --- | --- | --- | --- | --- |
| Bivalvia | 0.0843 | 0.0771 | 1.0933 | 0.1758 | 0.3144 | 0.1640 | 0.999 |
| Decapoda | 0.0835 | 0.0797 | 1.0477 | 0.1872 | 0.2926 | 0.3264 | 0.869 |
| Polychaeta | 0.0821 | 0.0816 | 1.0057 | 0.4834 | 0.4262 | 0.4860 | 0.999 |
| Oligochaeta | 0.0790 | 0.0808 | 0.9780 | 0.4734 | 0.4317 | 0.6397 | 1 |
| Gastropoda | 0.0501 | 0.0675 | 0.7426 | 0.1457 | 0.0883 | 0.7372 | 0.001 |
| Nematoda | 0.0487 | 0.0640 | 0.7620 | 0.1263 | 0.1089 | 0.8321 | 0.001 |
| Tanaidae | 0.0284 | 0.0534 | 0.5306 | 0.0638 | 0.0579 | 0.8872 | 0.001 |
| Hirudinea | 0.0276 | 0.0522 | 0.5279 | 0.0718 | 0.0469 | 0.9409 | 0.01 |
| Amphipoda | 0.0200 | 0.0472 | 0.4225 | 0.0276 | 0.0538 | 0.9797 | 0.999 |
| Coleoptera | 0.0104 | 0.0347 | 0.3010 | 0.0268 | 0.0152 | 1.0000 | 0.094 |

Contrast: November 2018–March 2019

| Taxa | average | SD | ratio | ava | avb | cumsum | *p* |
| --- | --- | --- | --- | --- | --- | --- | --- |
| Oligochaeta | 0.1049 | 0.0982 | 1.0676 | 0.4734 | 0.3671 | 0.1910 | 0.606 |
| Bivalvia | 0.1024 | 0.0946 | 1.0820 | 0.1758 | 0.3501 | 0.3776 | 0.23 |
| Polychaeta | 0.1004 | 0.0954 | 1.0526 | 0.4834 | 0.4949 | 0.5605 | 0.632 |
| Decapoda | 0.0847 | 0.0878 | 0.9652 | 0.1872 | 0.2443 | 0.7149 | 0.84 |
| Gastropoda | 0.0405 | 0.0657 | 0.6157 | 0.1457 | 0.0000 | 0.7886 | 0.005 |
| Nematoda | 0.0347 | 0.0591 | 0.5870 | 0.1263 | 0.0000 | 0.8518 | 0.01 |
| Amphipoda | 0.0319 | 0.0628 | 0.5082 | 0.0276 | 0.0976 | 0.9100 | 0.887 |
| Hirudinea | 0.0226 | 0.0504 | 0.4477 | 0.0718 | 0.0144 | 0.9511 | 0.046 |
| Tanaidae | 0.0176 | 0.0467 | 0.3775 | 0.0638 | 0.0000 | 0.9832 | 0.03 |
| Coleoptera | 0.0092 | 0.0352 | 0.2618 | 0.0268 | 0.0066 | 1.0000 | 0.126 |

Contrast: December 2018–January 2019

| Taxa | average | SD | ratio | ava | avb | cumsum | *p* |
| --- | --- | --- | --- | --- | --- | --- | --- |
| Oligochaeta | 0.1235 | 0.1127 | 1.0963 | 0.5705 | 0.4102 | 0.2409 | 0.001 |
| Polychaeta | 0.1116 | 0.1061 | 1.0520 | 0.4220 | 0.5378 | 0.4585 | 0.042 |
| Bivalvia | 0.0930 | 0.0931 | 0.9990 | 0.2075 | 0.2555 | 0.6398 | 0.964 |
| Decapoda | 0.0857 | 0.0915 | 0.9361 | 0.1911 | 0.2175 | 0.8069 | 0.798 |
| Amphipoda | 0.0373 | 0.0655 | 0.5691 | 0.0487 | 0.0988 | 0.8795 | 0.575 |
| Gastropoda | 0.0224 | 0.0550 | 0.4078 | 0.0674 | 0.0133 | 0.9233 | 0.691 |
| Nematoda | 0.0162 | 0.0416 | 0.3896 | 0.0462 | 0.0175 | 0.9548 | 0.97 |
| Hirudinea | 0.0150 | 0.0503 | 0.2987 | 0.0420 | 0.0086 | 0.9841 | 0.534 |
| Tanaidae | 0.0041 | 0.0231 | 0.1788 | 0.0059 | 0.0087 | 0.9922 | 0.992 |
| Coleoptera | 0.0040 | 0.0276 | 0.1453 | 0.0055 | 0.0082 | 1.0000 | 0.736 |

Contrast: December 2018–February 2019

| Taxa | average | SD | ratio | ava | avb | cumsum | *p* |
| --- | --- | --- | --- | --- | --- | --- | --- |
| Oligochaeta | 0.0985 | 0.0907 | 1.0863 | 0.5705 | 0.4317 | 0.1908 | 0.894 |
| Polychaeta | 0.0932 | 0.0858 | 1.0872 | 0.4220 | 0.4262 | 0.3714 | 0.939 |
| Bivalvia | 0.0906 | 0.0818 | 1.1076 | 0.2075 | 0.3144 | 0.5469 | 0.981 |
| Decapoda | 0.0881 | 0.0847 | 1.0399 | 0.1911 | 0.2926 | 0.7175 | 0.625 |
| Gastropoda | 0.0375 | 0.0649 | 0.5778 | 0.0674 | 0.0883 | 0.7901 | 0.007 |
| Nematoda | 0.0375 | 0.0609 | 0.6152 | 0.0462 | 0.1089 | 0.8627 | 0.007 |
| Amphipoda | 0.0249 | 0.0515 | 0.4839 | 0.0487 | 0.0538 | 0.9109 | 0.993 |
| Hirudinea | 0.0233 | 0.0551 | 0.4227 | 0.0420 | 0.0469 | 0.9561 | 0.031 |
| Tanaidae | 0.0173 | 0.0441 | 0.3916 | 0.0059 | 0.0579 | 0.9896 | 0.027 |
| Coleoptera | 0.0054 | 0.0258 | 0.2089 | 0.0055 | 0.0152 | 1.0000 | 0.506 |

Contrast: December 2018–March 2019

| Taxa | average | SD | ratio | ava | avb | cumsum | *p* |
| --- | --- | --- | --- | --- | --- | --- | --- |
| Oligochaeta | 0.1286 | 0.1150 | 1.1187 | 0.5705 | 0.3671 | 0.2422 | 0.001 |
| Polychaeta | 0.1146 | 0.1064 | 1.0769 | 0.4220 | 0.4949 | 0.4579 | 0.006 |
| Bivalvia | 0.1090 | 0.1007 | 1.0820 | 0.2075 | 0.3501 | 0.6631 | 0.004 |
| Decapoda | 0.0890 | 0.0932 | 0.9547 | 0.1911 | 0.2443 | 0.8307 | 0.511 |
| Amphipoda | 0.0372 | 0.0665 | 0.5599 | 0.0487 | 0.0976 | 0.9008 | 0.584 |
| Gastropoda | 0.0191 | 0.0502 | 0.3812 | 0.0674 | 0.0000 | 0.9368 | 0.894 |
| Hirudinea | 0.0165 | 0.0522 | 0.3161 | 0.0420 | 0.0144 | 0.9679 | 0.371 |
| Nematoda | 0.0120 | 0.0346 | 0.3457 | 0.0462 | 0.0000 | 0.9905 | 1 |
| Coleoptera | 0.0035 | 0.0237 | 0.1470 | 0.0055 | 0.0066 | 0.9970 | 0.808 |
| Tanaidae | 0.0016 | 0.0125 | 0.1255 | 0.0059 | 0.0000 | 1.0000 | 1 |

Contrast: January 2019–February 2019

| Taxa | average | SD | ratio | ava | avb | cumsum | *p* |
| --- | --- | --- | --- | --- | --- | --- | --- |
| Decapoda | 0.0946 | 0.0883 | 1.0722 | 0.2175 | 0.2926 | 0.1866 | 0.069 |
| Bivalvia | 0.0932 | 0.0812 | 1.1472 | 0.2555 | 0.3144 | 0.3703 | 0.984 |
| Oligochaeta | 0.0910 | 0.0805 | 1.1311 | 0.4102 | 0.4317 | 0.5498 | 1 |
| Polychaeta | 0.0904 | 0.0834 | 1.0839 | 0.5378 | 0.4262 | 0.7280 | 1 |
| Amphipoda | 0.0363 | 0.0627 | 0.5789 | 0.0988 | 0.0538 | 0.7996 | 0.676 |
| Nematoda | 0.0338 | 0.0608 | 0.5563 | 0.0175 | 0.1089 | 0.8662 | 0.002 |
| Gastropoda | 0.0279 | 0.0599 | 0.4661 | 0.0133 | 0.0883 | 0.9213 | 0.169 |
| Tanaidae | 0.0181 | 0.0455 | 0.3973 | 0.0087 | 0.0579 | 0.9569 | 0.001 |
| Hirudinea | 0.0151 | 0.0427 | 0.3541 | 0.0086 | 0.0469 | 0.9868 | 0.514 |
| Coleoptera | 0.0067 | 0.0332 | 0.2021 | 0.0082 | 0.0152 | 1.0000 | 0.338 |

Contrast: January 2019–March 2019

| Taxa | average | SD | ratio | ava | avb | cumsum | *p* |
| --- | --- | --- | --- | --- | --- | --- | --- |
| Oligochaeta | 0.1119 | 0.0989 | 1.1308 | 0.4102 | 0.3671 | 0.2255 | 0.023 |
| Bivalvia | 0.1104 | 0.0987 | 1.1181 | 0.2555 | 0.3501 | 0.4481 | 0.001 |
| Polychaeta | 0.1057 | 0.0974 | 1.0847 | 0.5378 | 0.4949 | 0.6611 | 0.159 |
| Decapoda | 0.0970 | 0.1002 | 0.9678 | 0.2175 | 0.2443 | 0.8567 | 0.002 |
| Amphipoda | 0.0478 | 0.0744 | 0.6430 | 0.0988 | 0.0976 | 0.9531 | 0.001 |
| Hirudinea | 0.0065 | 0.0309 | 0.2115 | 0.0086 | 0.0144 | 0.9663 | 1 |
| Nematoda | 0.0050 | 0.0266 | 0.1873 | 0.0175 | 0.0000 | 0.9764 | 1 |
| Coleoptera | 0.0050 | 0.0332 | 0.1499 | 0.0082 | 0.0066 | 0.9864 | 0.675 |
| Gastropoda | 0.0041 | 0.0275 | 0.1501 | 0.0133 | 0.0000 | 0.9947 | 1 |
| Tanaidae | 0.0026 | 0.0197 | 0.1325 | 0.0087 | 0.0000 | 1.0000 | 1 |

Contrast: February 2019–March 2019

| Taxa | average | SD | ratio | ava | avb | cumsum | *p* |
| --- | --- | --- | --- | --- | --- | --- | --- |
| Bivalvia | 0.0977 | 0.0833 | 1.1733 | 0.3144 | 0.3501 | 0.1918 | 0.757 |
| Decapoda | 0.0949 | 0.0883 | 1.0744 | 0.2926 | 0.2443 | 0.3781 | 0.066 |
| Polychaeta | 0.0944 | 0.0842 | 1.1203 | 0.4262 | 0.4949 | 0.5634 | 0.964 |
| Oligochaeta | 0.0910 | 0.0800 | 1.1375 | 0.4317 | 0.3671 | 0.7420 | 1 |
| Amphipoda | 0.0363 | 0.0635 | 0.5714 | 0.0538 | 0.0976 | 0.8133 | 0.674 |
| Nematoda | 0.0310 | 0.0591 | 0.5252 | 0.1089 | 0.0000 | 0.8742 | 0.011 |
| Gastropoda | 0.0252 | 0.0571 | 0.4414 | 0.0883 | 0.0000 | 0.9237 | 0.417 |
| Hirudinea | 0.0165 | 0.0446 | 0.3692 | 0.0469 | 0.0144 | 0.9560 | 0.351 |
| Tanaidae | 0.0162 | 0.0432 | 0.3743 | 0.0579 | 0.0000 | 0.9878 | 0.013 |
| Coleoptera | 0.0062 | 0.0307 | 0.2031 | 0.0152 | 0.0066 | 1.0000 | 0.408 |
